# Exploring Associations between Grey Matter Volume and Clinical High-Risk for Psychosis: A Transdiagnostic Study Utilizing the NAPLS-2 Risk Calculator in the PRONIA Cohort

**DOI:** 10.1192/j.eurpsy.2024.572

**Published:** 2024-08-27

**Authors:** L.-M. Neuner, L. Hahn, J. Kambeitz, R. K. Salokangas, J. Hietala, A. Bertolino, S. Borgwardt, P. Brambilla, R. Upthegrove, S. J. Wood, R. Lencer, E. Meisenzahl, P. Falkai, T. D. Cannon, N. Koutsouleris

**Affiliations:** ^1^Department of Psychiatry and Psychotherapy, LMU University Hospital, Ludwig-Maximilians-Universität, Munich; ^2^Department of Psychiatry and Psychotherapy, University of Cologne, Cologne, Germany; ^3^Department of Psychiatry, University of Turku, Turku, Finland; ^4^Department of Basic Medical Science, Neuroscience and Sense Organs, University of Bari Aldo Moro, Bari, Italy; ^5^Department of Psychiatry and Psychotherapy, University of Lübeck, Lübeck, Germany; ^6^Department of Psychiatry (Psychiatric University Hospital, UPK), University of Basel, Basel, Switzerland; ^7^Department of Neurosciences and Mental Health, Fondazione IRCCS Ca’ Granda Ospedale Maggiore Policlinico; ^8^Department of Pathophysiology and Transplantation, University of Milan, Milan, Italy; ^9^Institute of Mental Health, University of Birmingham; ^10^Early Intervention Service, Birmingham Women’s and Children’s NHS Foundation Trust; ^11^School of Psychology, University of Birmingham, Birmingham, United Kingdom; ^12^Centre for Youth Mental Health, University of Melbourne; ^13^Orygen, Melbourne, Australia; ^14^Institute for Translational Psychiatry, University Münster, Münster; ^15^Department of Psychiatry and Psychotherapy, Medical Faculty, Heinrich Heine University, Düsseldorf; ^16^Max-Planck Institute of Psychiatry, Munich, Germany; ^17^Department of Psychiatry; ^18^Department of Psychology, Yale University, New Haven, Connecticut, United States; ^19^Institute of Psychiatry, Psychology And Neurosciences, King’s College London, London, United Kingdom

## Abstract

**Introduction:**

The clinical high-risk state for psychosis (CHR) is associated with alterations in grey matter volume (GMV) in various regions such as the hippocampus (Vissink *et al.* BP:GOS 2022; 2(2) 147-152). Within the scope of the North American Prodrome Longitudinal Study (NAPLS-2; Cannon *et al.* AM J Psychiatry 2016; 173(10), 980-988), a publicly available risk calculator based on clinical variables was developed to assess the likelihood of individuals to transition to psychosis within a 2-year period.

**Objectives:**

In the current study, we aim to examine the association between GMV and NAPLS-2 risk scores calculated for individuals with CHR and recent-onset depression (ROD), taking a transdiagnostic approach on the transition to psychosis.

**Methods:**

The sample consisted of 315 CHR (*M* = 23.85, *SD* = ± 5.64; female: 164) and 295 ROD (*M* = 25.11, *SD* = ± 6.21; female: 144) patients from the multi-site Personalised Prognostic Tools for Early Psychosis Management (PRONIA) Study (Koutsouleris *et al*. JAMA Psychiatry 2018; 57(11), 1156-1172). Risk scores were calculated using the six clinical and neurocognitive variables included in the NAPLS-2 risk calculator that were significant for predicting psychosis. Further, we derived smoothed GMV maps from T1-weighted structural magnetic resonance imaging using a full width at half maximum kernel size of 8 mm. We employed a multiple regression design in SPM12 to examine associations between risk scores and GMV. On the whole-brain level, we calculated permutation-based threshold-free cluster enhancement (TFCE) contrasts using the TFCE toolbox. Additionally, we calculated t-contrasts within a region-of-interest (ROI) analysis encompassing the hippocampus. All results were thresholded at p < 0.05 with family wise error correction to address multiple comparisons.

**Results:**

Our analysis revealed that linear GMV increases in the right middle and superior frontal gyrus (k_E_= 2726 voxels) were significantly associated with higher risk for psychosis transition within two years (see figure 1, highlighted in blue). In the ROI analysis, we found a significant negative linear association between GMV decreases in the left hippocampus (k_E_ = 353 voxels) and higher risk for psychosis transition (see figure 1, highlighted in red).

**Image:**

**Figure:**
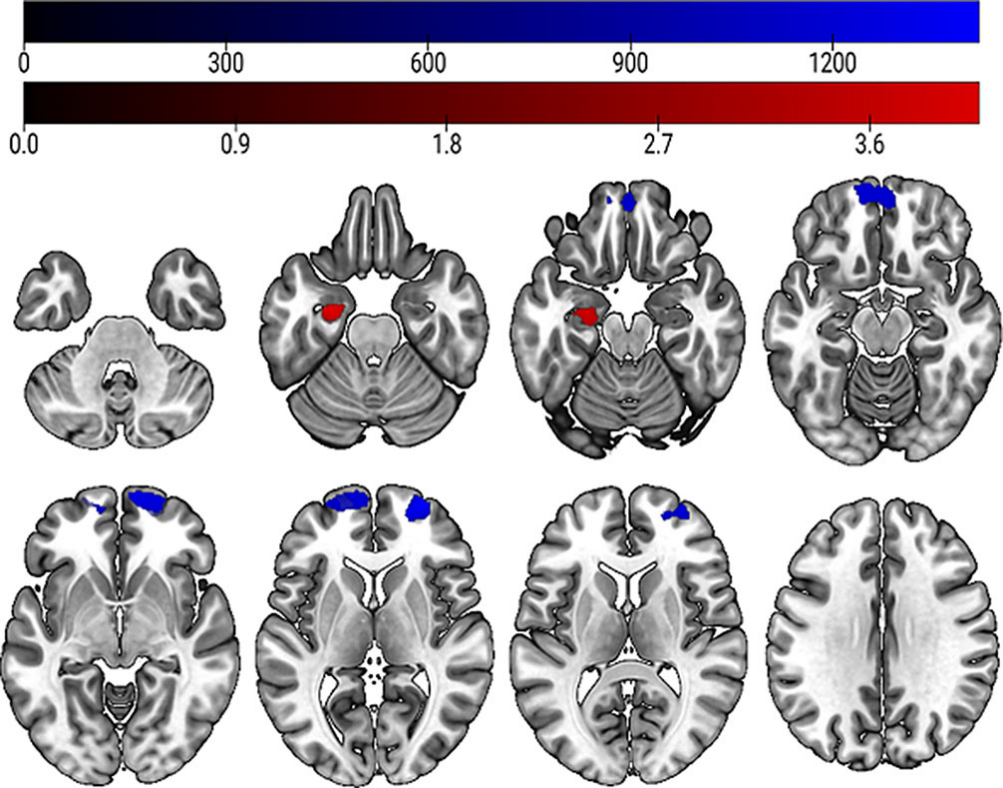

**Conclusions:**

GMV reductions in the hippocampus have frequently been observed in CHR and psychosis patients (Vissink *et al.* BP:GOS 2022; 2(2) 147-152), therefore our results further highlight the crucial role of this region in the progression of the disease. There is limited evidence on GMV increases in CHR patients. However, the GMV increase we found in the frontal pole may reflect compensatory mechanisms of the brain in the development of psychosis. In addition, we were able to provide biological validation of the NAPLS-2 risk calculator and its assessment of risk for transition to psychosis.

**Disclosure of Interest:**

None Declared

